# Identification of Cardiovascular Risk Components in Urban Chinese with Metabolic Syndrome and Application to Coronary Heart Disease Prediction: A Longitudinal Study

**DOI:** 10.1371/journal.pone.0084204

**Published:** 2013-12-17

**Authors:** Zhenxin Zhu, Yanxun Liu, Chengqi Zhang, Zhongshang Yuan, Qian Zhang, Fang Tang, Haiyan Lin, Yongyuan Zhang, Longjian Liu, Fuzhong Xue

**Affiliations:** 1 Department of Epidemiology and Biostatistics, School of Public Health, Shandong University, Jinan, Shandong, China; 2 Health Management Center, Shandong Provincial QianFoShan Hospital, Jinan, Shandong, China; 3 Center for Health Management, Provincial Hospital affiliated to Shandong University, Jinan, Shandong, China; 4 Department of Epidemiology and Biostatistics, Drexel University School of Public Health, Philadelphia, Pennsylvania, United States of America; VCU, United States of America

## Abstract

**Background:**

Metabolic syndrome (MetS) is proposed as a predictor for cardiovascular disease (CVD). It involves the mechanisms of insulin resistance, obesity, inflammation process of atherosclerosis, and their complex relationship in the metabolic network. Therefore, more cardiovascular risk-related biomarkers within this network should be considered as components of MetS in order to improve the prediction of CVD.

**Methods:**

Factor analysis was performed in 5311 (4574 males and 737 females) Han Chinese subjects with MetS to extract CVD-related factors with specific clinical significance from 16 biomarkers tested in routine health check-up. Logistic regression model, based on an extreme case-control design with 445 coronary heart disease (CHD) patients and 890 controls, was performed to evaluate the extracted factors used to identify CHD. Then, Cox model, based on a cohort design with 1923 subjects followed up for 5 years, was conducted to validate their predictive effects. Finally, a synthetic predictor (SP) was created by weighting each factor with their risks for CHD to develop a risk matrix to predicting CHD.

**Results:**

Eight factors were obtained from both males and females with a similar pattern. The AUC to classify CHD under the extreme case-control suggested that *SP* might serve as a useful tool in identifying CHD with 0.994 (95%CI 0.984-0.998) for males and 0.998 (95%CI 0.982-1.000) for females respectively. In the cohort study, the AUC to predict CHD was 0.871 (95%CI 0.851-0.889) for males and 0.899 (95%CI 0.873-0.921) for females, highlighting that *SP* was a powerful predictor for CHD. The *SP*-based 5-year CHD risk matrix provided as convenient tool for CHD risk appraisal.

**Conclusions:**

Eight factors were extracted from sixteen biomarkers in subjects with MetS and the *SP* adds to new insights into studies of prediction of CHD risk using data from routine health check-up.

## Introduction

Metabolic syndrome (MetS) is a public health challenge because of its high prevalence and association with the risk of cardiovascular disease (CVD) [1,2] and type 2 diabetes [3,4]. Several studies have applied MetS as a marker to predict the development of CVD at the population level, however, few studies have been conducted in Asian populations. According to the criteria recommended by the Diabetes Branch of Chinese Medical Association [5], MetS encompasses a cluster of metabolically related CVD risk factors: being overweight or obese, high blood pressure, dyslipidemia, and hyperglycemia. This criterion is slightly different from the international definition of MetS [6]. In pathogenesis, MetS, defined by either Chinese or international criteria, is defined using factors including obesity, diabetes, hypertension, and dyslipidemia. Because these factors are involved in the mechanisms of insulin resistance, and the process of inflammation and atherosclerosis, this complex relationship has been suggested in study as the metabolic network of MetS [7]. Therefore, a generalized definition of MetS could be extended using multiple components within this network. Several studies have suggested that the definition of MetS may further include microalbuminuria, proinflammatory cytokines, prothrombotic & fibrinolytic factors, and oxidative stress [8,9]. However, the structure and inclusion of MetS components are inconclusive [10]. Some studies found three or four factors, underlying the overall correlation between metabolic variables [11,12], while in recent years, some researchers verified a single-factor model that can represent MetS [13,14]. The different patterns of MetS components resulted from differences in data availability, the number of biomarkers incorporated into specific models, and studies with specific purposes. In the present study, we aimed to select several cardiovascular risk biomarkers involved in the above metabolic network using robust bio-statistical modeling technique to develop a MetS related synthetic predictor (SP) for classifying subjects with or without CVD, and to predict high risk of CVD using data from a large-scale routine health check-up sample among urban Chinese residents.

## Materials and Methods

### Ethics Statement

This study was approved by the Ethics Committee of School of Public Health, Shandong University, and all participants were informed by written consent to participate in this study. The data was de-identified before it was provided to us. The data can’t be shared with researchers upon request because while we cooperate with the hospital and have the right to use the data, the hospital is reluctant to let us share the data.

### Study population

The study population includes a cohort of all participants who received routine health check-ups from 2005 to 2010 at the Center for Health Management of Shandong Provincial QianFoShan Hospital, and the Health Examination Center of Shandong Provincial Hospital. These two hospitals are affiliated teaching hospitals of the Shandong University, Jinan, Shandong province, China. Participants are urban residents living in Jinan city, the capital of Shandong province, and they were primarily employees. Participants who had completed physical examinations and the measurements of the study of biomarkers were included (n=28200). Of them, 5311 were classified having MetS (4574 males and 737 females) at their first health check-up using the criteria of the Chinese Medical Association. In the study, we used CHD as the study outcome, because MetS is suggested as an independent predictor for CHD [15-17]. Of 5311 MetS subjects, 445 cases (292 males and 153 females) had CHD diagnosed by physicians. Controls (n=890, males=584, and females=306) were randomly selected from the individuals without any components of MetS in the present case-control study design. Of 28200, 1923 participants (1263 males and 660 females) who had no CHD at baseline and completed a 5-year follow-up were included in the present cohort study design. At the end of the follow-up period, 134 incident CHD cases (90 males and 44 females) were diagnosed and the cumulative incidence rate was 6.97% (7.13% in males and 6.67% in females) (Table S1). 

### Selection of biomarkers and measurements

In the present study, according to the complex relationship and the concept of metabolic network [7], we selected 16 biomarkers from the routine health check-up. They included non-alcoholic fatty liver (NAFLD), body mass index (BMI), systolic blood pressure (SBP), diastolic BP (DBP), total cholesterol (TC), low-density lipoprotein cholesterol (LDL-C), triglycerides (TG), high-density lipoprotein cholesterol (HDL-C), hemoglobin (Hb), hematokrit (HCT), alanine aminotransferase (ALT), gamma-glutamyl transpeptidase (GGT), serum uric acid (SUA), white blood cell (WBC) count, serum creatinine (CREA), and fasting blood-glucose (FBG). Among them, BMI, SBP, DBP, TG, HDL-C and FBG have been included in the traditional definition of MetS. Also, the others had been confirmed to meet the above criterion in longitudinal cohort studies, including NAFLD [18], TC [19-22], LDL-C [23], Hb/HCT [24], ALT/GGT [25-29], SUA [30-34], WBC count [35-39], and CREA [40]. 

All participants received a general health questionnaire survey, anthropometric measurement, and routine blood sample tests. The questionnaire covered demographic background and medical history. Anthropometric measurements included height, weight, and BP; both height and weight were measured with light clothing without shoes. BMI was calculated by weight/height^2^ (kg/m^2^). BP was measured on the right arm from a sitting position after a 5-minute rest. Blood samples were drawn after overnight fasting (>8 hours) for laboratory tests. All lab tests were conducted by certified experimental specialists using standard protocols at the hospital’s Department of Laboratory. WBC, Hb and HCT were measured using SYSMEX XE-2100 automatic whole blood count system. FBG was measured by enzymic method, TC & GGT by enzymic colorimetric method, SUA & TG by colorimetric method, HDL-C & LDL-C by direct method, ALT by the criteria of International Federation of Clinical Chemistry (IFCC), and CREA by picric acid method using Roche Cobas 8000 Automatic Biochemical Analyzer. NAFLD was diagnosed by abdominal ultrasonography as brightness of the liver and a diffusely echogenic change in the liver parenchyma, with participants who were diagnosed alcoholic fatty liver disease, infected hepatitis virus (Hepatitis B antigen or hepatitis C antibody positive), and other causes of steatosis (Wilson disease) excluded. Based on the diagnostic criteria recommended by the Diabetes Branch of Chinese Medical Association[5], MetS was defined as presence of three or more of the following four risk factors: 1) overweight or obesity (BMI≥25.0 Kg/M^2^); 2) hypertension (SBP≥140mmHg, DBP≥90mmHg or those with history of hypertension); 3) hyperglycemia (FPG≥6.1mmol/L or 2h plasma glucose≥7.8 mmol/L, or those with history of diabetes); 4) dyslipidemia (TG≥1.7 mmol/L, or HDL-C<0.9 mmol/L in male and HDL-C<1.0 mmol/L in female). CHD was diagnosed by physicians using the World Health Organization’s criteria (symptoms plus either diagnostic ECG changes or elevated levels of cardiac enzymes) [41].

### Strategy of the development of synthetic predictor

In the study, the orthogonal exploratory factor analysis (EFA), a standard method to identify patterns of MetS, was used to extract cardiovascular risk-related factors from the above 16 manifest biomarkers in the MetS population. After EFA, the clinical significance of each latent factor was named. Furthermore, the logistic regression discrimination (LRD) model based on an extreme case-control design was performed to evaluate their discriminant effects for CHD, and the Cox proportional hazard prediction model based on a cohort design was further conducted to validate their predictive effects. Finally, *SP* was created by weighting each factor with their risks for CHD for developing a risk matrix to predict CHD in the practice of routine health check-up.

### Statistical analysis

#### Descriptive analysis

For patients with MetS, student *t* test (for continuous variables) and the χ^2^ test (for categorical variables) were used to test the significant differences of the sixteen biomarkers between males and females. The difference in the prevalence of the four basic components (obesity, hypertension, dyslipidemia and hyperglycemia) and their combination between males and females were tested by χ^2^ test.

#### Steps of the development of synthetic predictor

EFA with principal component algorithm and varimax rotation from correlation matrix was performed to extract independent factors of MetS from above 16 manifest biomarkers for male and female MetS groups respectively. The criteria for retaining factors were set up as eigenvalue>1 as well as accounting for 70% of the total variation. Only variables that shared at least 15% of the factor variance, corresponding to a factor loading of at least 0.40 were used for further analytical interpretation [42,43]. After EFA, the clinical significance of each latent factor was named, and *SP* was created using a weighted approach:*SP*=*γ*_1_ *F*_1_+*γ*_2_ *F*_2_+⋯+*γ*_*k*_ *F*_*k*_, where *F*_1_,*F*_2_,⋯*F*_*k*_ were the extracted independent factors with specific clinical significance from the 16 manifest biomarkers, and *γ*_1_,*γ*_2_,⋯*γ*_*k*_denoted their risks to CHD, which were partial regression coefficients in LRD and Cox regression models described below.

The LRD, on the basis of the extreme case-control design, was performed using the model, 

P=[(exp(Β))/(1+exp(Β))] (1)

where *P* was the probability of CHD. *Β* denoted the discriminant vector estimated by logistic regression, where Β=*β*_0_+*β*_1_ *age*+*γ*_1_ *F*_1_+*γ*_2_ *F*_2_+⋯+*γ*_*k*_ *F*_*k*_ or Β=*β*_0_+*β*_1_ *age*+*γSP*. 

The Cox model, on the basis of cohort study design, was conducted to validate the predictive effects using the formula below, 

$$P(t)=1-S(t)=1-\exp(-\int_{\text{0}}^{t}h_{0}(u)\exp(\text{Β})du)$$ (2)

where *P*(*t*) was the predictive probability of CHD at year *t*. *Β* denoted the predictor vector estimated by Cox regression, where *Β*=*β*_1_ *age*+*γ*_1_ *F*_1_+*γ*_2_ *F*_2_+⋯+*γ*_*k*_ *F*_*k*_ or *Β*=*β*_1_ *age*+*γSP*. The average probability at time *t* of *j* ^th^ age (${\bar{P}}_{j}(t)$), which approximately referred to the baseline hazard rate of general population, was calculated by model (2) through${\bar{\text{Β}}}_{j}=\beta_{1}age_{j}+\gamma {\bar{SP}}_{j}$, where ${\bar{SP}}_{j}$ was the mean of *SP* in *j* ^th^ age from the general population. Therefore, the absolute risk (AR) of an individual with age *j* at time *t* was calculated by *P*_*j*_(*t*), the excess absolute risk (EAR) by $P_{j}(t)-{\bar{P}}_{j}(t)$_,_ and the relative absolute risk (RAR) by $P_{j}(t)/{\bar{P}}_{j}(t)$_._

Receiver operating characteristic (ROC) curve was used to evaluate the discriminant effect for LRD model (1), and to validate their predictive effect for Cox model (2). The area under the curve (AUC) for the ROC analysis together with sensitivity, specificity, and cutoff of *P* value was calculated using MedCalc software for ROC curve analysis[44].

In practice, to calculate the AR of CHD for a person using model (2), the *SP* needed to be turned to its original expression with the raw 16 biomarkers by,

$$SP=\alpha_{0}+\alpha_{1}NAFLD+\alpha_{2}BMI+\cdots +\alpha_{16}HCT$$ (3)

where *α*_1_,*α*_2_,⋯*α*_16_ denoted the weights of 16 biomarkers after destandardization with mean (*μ*) and standard deviation (*σ*) from the large sample in the routine health check-up database.

Finally, we calculated *SP* of 28200 individuals to identify the high risk individuals with optimal cutoff provided by ROC curve for predicting risk of CHD.

All data analyses were conducted for males and females separately. The risk matrix for AR and RAR were depicted using ArcGIS 9.1, and all statistical analysis of predictive models was performed using SAS 9.1. A two-sided p value <0.05 was considered as having statistical significance.

## Results

The prevalence of MetS in the study sample was 18.83%. Table 1 shows the distribution of age and 16 biomarkers between males and females with MetS. All variables were significantly different between genders. Of these biomarkers, BMI, DBP, SUA, TG, WBC count, ALT, GGT, CREA, Hb, HCT and prevalence of NAFLD were higher in males than in females, while SBP, FBG, TC, HDL-C and LDL-C were higher in females than in males. Table S2 shows significant correlations in most biomarkers.

**Table 1:** Distribution of age and the sixteen biomarkers between male and female metabolic syndrome groups.

	Male(n=4574)		Female(n=737)		*P* value
	mean	SD	mean	SD	
Age (years)	50.85	11.96	60.65	11.19	<0.001
Body mass index (kg/m^2^)	28.13	2.57	27.88	3.04	0.03
Systolic blood pressure (mmHg)	143.00	17.45	150.45	20.51	<0.001
Diastolic blood pressure (mmHg)	87.24	12.00	81.65	12.09	<0.001
Serum uric acid (umol/L)	383.15	81.91	307.91	70.95	<0.001
Fasting blood-glucose (mmol/L)	6.48	1.86	6.67	1.94	0.01
Total cholesterol (mmol/L)	5.56	1.05	5.97	1.14	<0.001
Triglycerides (mmol/L)	2.94	2.33	2.32	1.42	<0.001
High-density lipoprotein cholesterol (mmol/L)	1.22	0.34	1.35	0.37	<0.001
Low-density lipoprotein cholesterol (mmol/L)	3.23	0.77	3.45	0.79	<0.001
White blood cell count (10^9^/L)	7.07	1.68	6.90	1.64	0.01
Alanine aminotransferase (U/L)	31.21	22.39	22.66	16.69	<0.001
Gamma-glutamyl transpeptidase (U/L)	54.86	52.15	26.67	29.69	<0.001
Serum creatinine (umol/L)	88.13	13.69	72.63	16.08	<0.001
Hemoglobin (g/L)	157.72	10.45	137.99	11.17	<0.001
Hematokrit (%)	45.75	2.98	41.01	2.87	<0.001
Non-alcoholic fatty liver (n/%)	2875	62.86	417	56.58	<0.001

SD denotes standard deviation.

Table S3 and Figure S1 showed that the prevalence of 4 basic components (BMI≥25 kg/m^2^, high BP, hyperglycemia, and dyslipidemia), and their combinations were significantly different.

Table 2 shows the explained variance, cumulative variance and loadings of the first eight factors. The results suggested that 75.07% and 75.49% of total variance were explained by the first eight factors for males and females respectively. Following the criteria of analytical interpretation mentioned in the statistical analysis section, eight independent factors with their specific clinical significances were retained and named for two groups respectively. For the male group, the first factor was named erythrocyte viscosity factor (EVF) and contributed by Hb & HCT, following lipid viscosity factor (LVF) by TC & LDL-C, lipid metabolism factor (LMF) by TG & HDL-C, blood pressure factor (BPF) by SBP & DBP, hepatic enzyme factor (HEF) by ALT & GGT, glucose metabolism factor (GMF) by FBG & SUA & CREA, fat accumulation factor (FAF) by NAFLD & BMI, and inflammation response factor (IRF) by WBC count & CREA. For the female group, the first was named LVF and contributed by TC & HDL-C & LDL-C, following EVF by Hb & HCT, HEF by ALT & GGT, BPF by SBP & DBP & BMI, IRF by SUA & CREA & WBC count, LMF by TG & HDL-C, GMF by FBG & WBC count, and FAF by FLD & BMI. These suggested that the patterns of factors were similar between males and females, though their ranks with descending order of explained variance were slightly different. Table S4 shows the standardized scoring coefficients of each factor for male and female groups.

**Table 2:** Factor loadings by principal component analysis with varimax rotation on 16 routine health check-up biomarkers in MetS patients.

	**Male** (n=4574)								**Female** (n=737)							
	Factor1	Factor2	Factor3	Factor4	Factor5	Factor6	Factor7	Factor8	Factor1	Factor2	Factor3	Factor4	Factor5	Factor6	Factor7	Factor8
	EVF	LVF	LMF	BPF	HEF	GMF	FAF	IRF	LVF	EVF	HEF	BPF	IRF	LMF	GMF	FAF
NAFLD	0.076	0.026	0.113	-0.061	-0.123	0.075	***0.839***	-0.115	0.025	0.054	-0.001	-0.087	0.010	0.049	-0.032	***0.883***
BMI	-0.048	-0.012	-0.171	0.194	0.298	-0.016	***0.596***	0.207	-0.070	-0.120	0.125	***0.424***	0.019	-0.072	0.116	***0.506***
SBP	-0.074	0.019	-0.021	***0.880***	-0.090	-0.051	-0.021	0.081	0.138	0.011	-0.107	***0.764***	0.066	-0.093	0.153	-0.025
DBP	0.213	-0.006	0.081	***0.822***	0.140	0.109	0.087	-0.066	-0.099	0.118	0.009	***0.807***	-0.095	0.083	-0.206	0.008
SUA	-0.029	0.002	0.165	0.002	0.153	***0.750***	0.148	0.180	-0.006	0.072	0.110	-0.075	***0.798***	-0.028	-0.168	0.171
FBG	-0.051	0.061	0.200	-0.032	0.048	***-0.736***	0.038	0.198	0.131	0.035	-0.002	-0.085	-0.176	0.023	***0.864***	0.043
Total cholesterol	0.071	***0.878***	0.383	0.023	0.082	-0.030	0.009	0.024	***0.923***	0.127	0.037	0.017	0.004	0.268	0.050	-0.005
Triglycerides	0.037	-0.078	***0.845***	-0.067	0.137	-0.013	0.115	0.095	-0.004	0.005	0.021	-0.093	0.038	***0.897***	0.045	0.094
HDL-C	-0.020	0.295	***0.744***	0.143	0.006	-0.039	-0.131	-0.113	***0.471***	0.088	0.043	0.125	-0.050	***0.586***	0.047	-0.178
LDL-C	0.079	***0.961***	-0.113	-0.009	0.047	-0.015	0.015	0.029	***0.921***	0.093	0.058	-0.023	0.039	-0.109	0.009	0.027
WBC count	0.168	-0.007	0.016	-0.007	0.049	-0.119	0.088	***0.787***	-0.162	0.175	0.039	0.182	***0.416***	0.113	***0.530***	-0.041
ALT	0.110	0.034	-0.065	-0.020	***0.833***	-0.015	0.067	0.034	0.058	0.156	***0.839***	-0.044	-0.028	-0.005	-0.044	0.143
GGT	0.047	0.088	0.303	0.054	***0.740***	0.088	-0.041	-0.094	0.035	0.016	***0.881***	-0.016	0.028	0.048	0.056	-0.068
Serum creatinine	-0.100	0.093	-0.032	0.059	-0.197	**0.415**	-0.183	***0.562***	0.063	-0.146	-0.105	0.021	***0.758***	0.018	0.068	-0.120
Hemoglobin	***0.951***	0.059	0.022	0.067	0.095	-0.003	0.028	0.034	0.108	***0.958***	0.066	0.045	-0.016	0.040	0.056	0.007
Hematokrit	***0.950***	0.075	0.004	0.041	0.064	0.006	0.019	0.091	0.119	***0.949***	0.114	0.046	-0.034	0.017	0.062	-0.011
% Variance explained	12.09	11.36	10.25	9.57	9.25	8.25	7.31	6.98	12.71	12.28	9.73	9.37	8.98	7.96	7.25	7.19
Cumulative variance	12.09	23.45	33.71	43.28	52.53	60.78	68.09	75.07	12.71	25.00	34.73	44.10	53.08	61.04	68.29	75.49

Factors were named as erythrocyte viscosity factor (EVF), lipid viscosity factor (LVF), lipid metabolism factor (LMF), blood pressure factor (BPF), hepatic enzyme factor (HEF), glucose metabolism factor (GMF), fat accumulation factor (FAF), and inflammation response factor (IRF). MetS indicates metabolic syndrome, NAFLD, non-alcoholic fatty liver; BMI, body mass index; SBP, systolic blood pressure; DBP, diastolic blood pressure; SUA, serum uric acid; FBG, fasting blood-glucose; HDL-C, high-density lipoprotein cholesterol; LDL-C, low-density lipoprotein cholesterol; WBC, white blood cell; ALT, alanine aminotransferase; and GGT, gamma-glutamyl transpeptidase.

**Figure 1:**
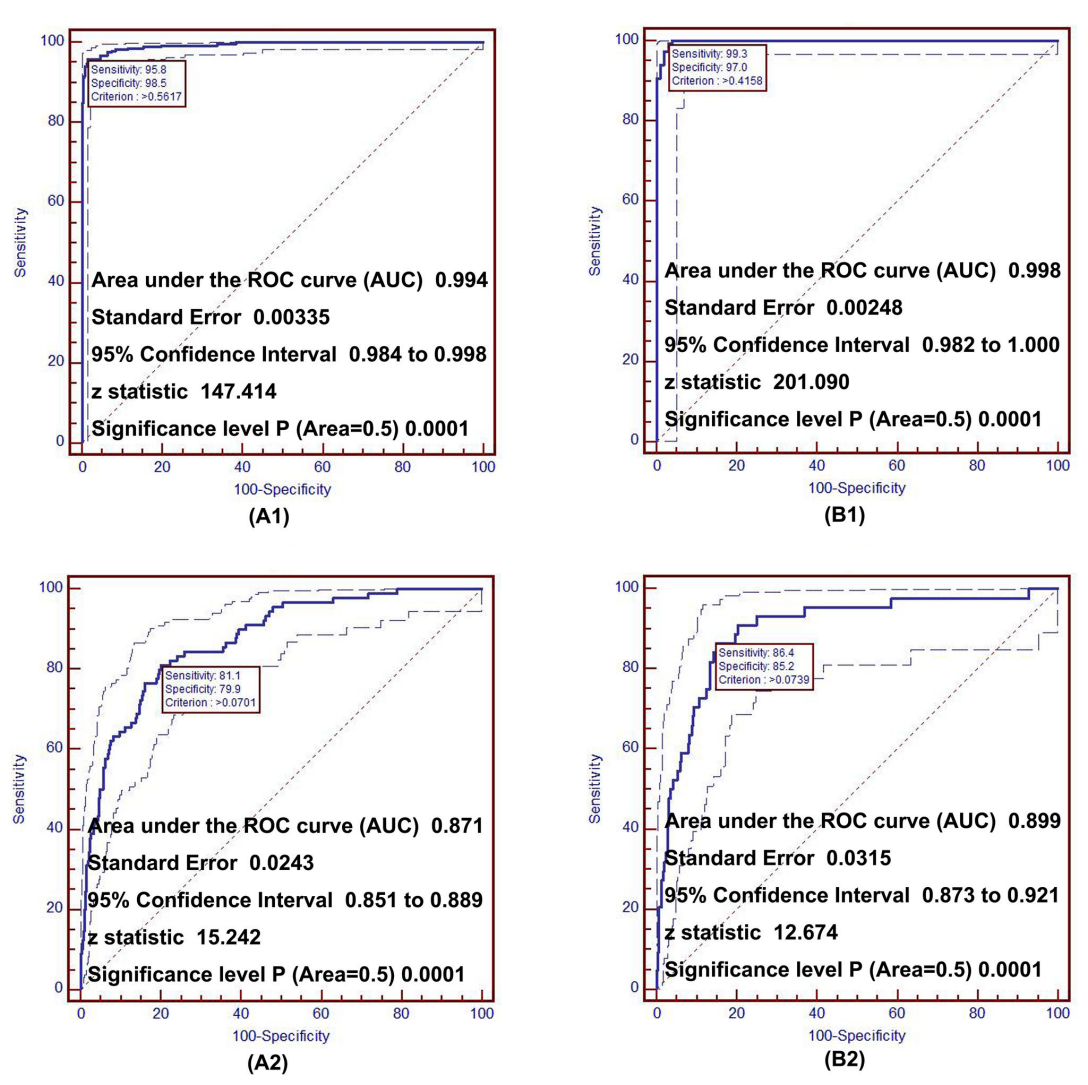
ROC curve for discrimination and prediction of coronary heart disease. A1 and B1 show the discriminative curve under extreme case-control design for male and female respectively; A2 and B2 show the predictive curve under cohort design for male and female respectively. ROC, receiver operating characteristic.

Figure 1_A1 and Figure 1_B1 depict the AUC with sensitivity, specificity, and the cut-off points of *P* value (criterion) by LRD model from the extreme case-control design using age and *SP* as discriminant factors. 

The AUC was 0.994 (95%*CI* 0.984-0.998) in males, and 0.998 (95%*CI* 0.982-1.000) in females, suggesting that *SP* had a good performance for identifying subjects with or without CHD.

Figures 1_A2 and 1_B2 depicts the AUC with the study parameters to predict 5-year risk of CHD by Cox model from the cohort study design using age and *SP*. AUC was 0.871 (95%*CI* 0.851-0.889) in males, and 0.899 (95%*CI* 0.873-0.921) in females. 

**Figure 2:**
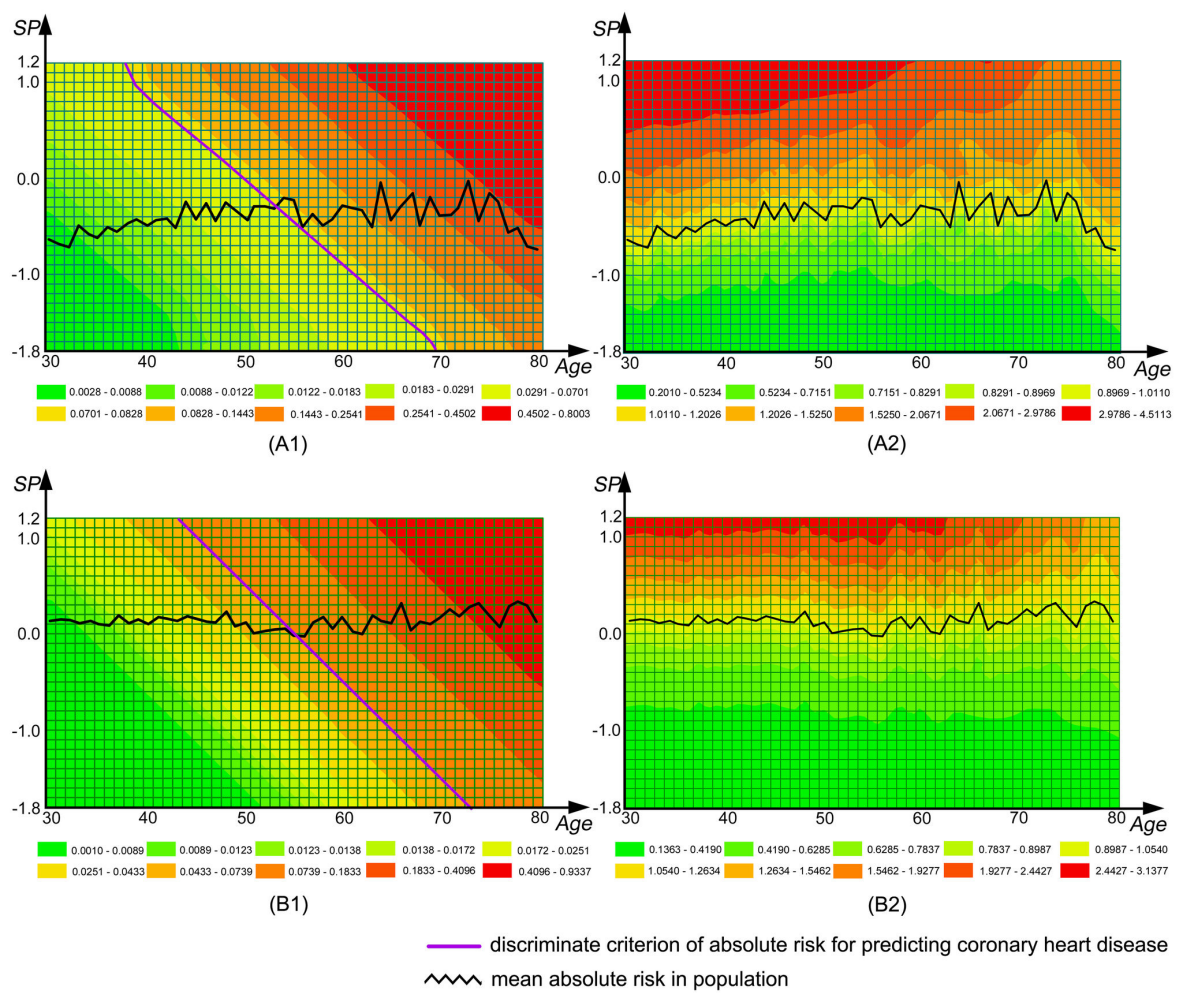
The 5-year risk matrix for risk appraisal of coronary heart disease by gender. A1 and B1 are absolute risk matrix of male and female respectively. A2 and B2 are relative absolute risk matrix of male and female respectively. *SP*, synthetic predictor. For male: *SP* = 0.2226NAFLD + 0.0749BMI + 0.0081SBP + 0.0103DBP - 0.0011SUA + 0.0422FBG - 0.0863TC - 0.0920TG - 0.6562HDL-C + 0.0090LDL-C + 0.0045WBC count + 0.0065ALT + 0.0009GGT - 0.0076CREA - 0.0080Hb - 0.0312HCT + 0.8816. For female: *SP* = 0.3974NAFLD + 0.0267BMI + 0.0118SBP + 0.0168DBP + 0.0012SUA - 0.2089FBG - 0.1421TC - 0.3229TG - 0.9395HDL-C - 0.0544LDL-C - 0.0421WBC count - 0.0395ALT - 0.0461GGT + 0.0087CREA + 0.0022Hb - 0.0015HCT + 0.8739.

Figure 2 shows the 5-year AR matrix and RAR matrix for CHD by gender (A1 and A2 indicating AR and RAR matrices for males, and B1 and B2 for females). These matrices provide a simple tool for conducting CHD prediction in health management and clinical practice. For example, a male aged 40-years-old and having AR of 0.082 has an EAR of 0.064 (0.082-0.018) and RAR of 4.56 (0.082/0.018), while a male aged 60-years-old and having AR of 0.082, has the EAR of -0.043 (0.082-0.125) and RAR of 0.656 (0.082/0.125). These show that although their predictive probabilities for CHD over 5 years are the same, the CHD risk of the younger male is higher than the average risk of the same age, almost 4.56 times that of his peers, indicating that changes in lifestyle and social intervention strategies are needed for him. Alternatively the older male has a lower CHD risk compared to his peers, only 65.6% of the average risk of 60-year-old population, indicating that he has a good health status compared with his peers.

**Figure 3:**
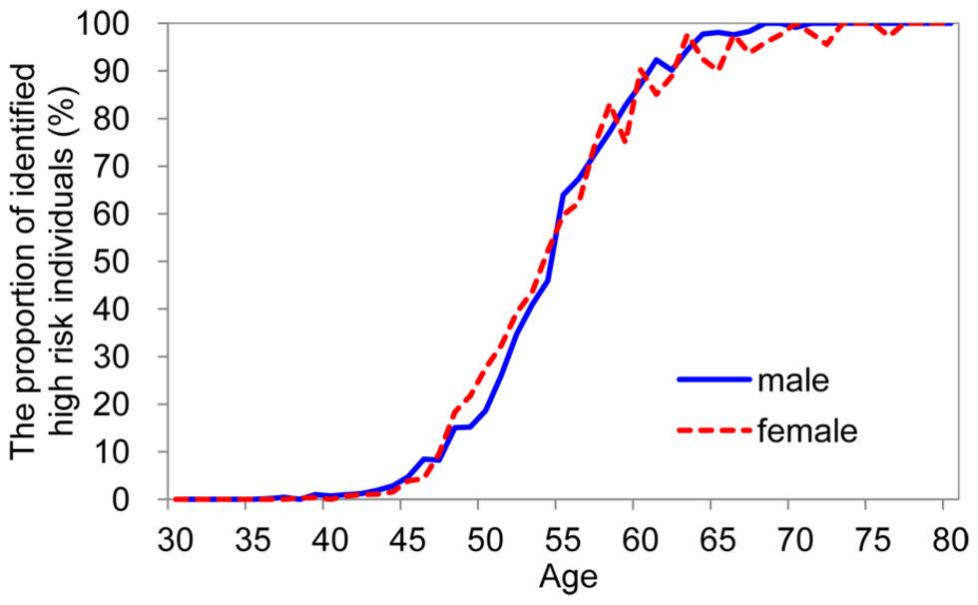
The coronary heart disease risk appraisal result of 28200 individuals in routine health check-up system.

Figure 3 shows the proportion of identified individuals with high risk of CHD among the total study sample (n=28200) using the cutoff point of AR with 0.0701 for male and 0.0739 for female as discriminate criterion (see Figures 1_A2 and 1_B2). The proportion of identified high risk individuals showed an S curve with age. For example, people at age 65 are at high risk of CHD (>90% for both genders) in the next 5 years. .

## Discussion

The present study is the first to apply a large population-based data sample from routine health check-ups in an urban resident sample in China. The study extended previous studies by adding new biomarkers to the network of MetS. The main findings suggest that a weighted *SP* developed from 8 latent variables are able to classify patients with and without CHD, and to predict subjects who are at high risk of CHD in 5 years.

### The components of MetS

Clinical use of criteria for MetS is primarily to predict risk of CVD and diabetes. MetS was commonly defined by the presence of obesity, diabetes, hypertension, and dyslipidemia, and these factors were involved in the mechanisms of insulin resistance, inflammation, and atherosclerosis [7]. This complex relationship can be considered as the metabolic network of MetS. Therefore, other cardiovascular risks related to this network should be included in the concept of MetS in order to predict risk of CVD and diabetes more precisely. Several studies have suggested that NAFLD [45,46], SUA [47], microalbuminuria, proinflammatory cytokines, prothrombotic and fibrinolytic factors, and oxidative stress [8,9] should be part of the components of MetS. However, the inclusion of extended biomarkers may be differ by study populations, and the availability of measurements. For example, data from routine health check-ups, the generalized concept of MetS should be extended using biomarkers, which are routinely measured with simple, inexpensive and standardized approaches. In this paper, we extended previous studies using factor analysis to identify 8 factors from 16 biomarkers they are measured in routine health check-up. Of 8 factors, EVF, LMF, HEF, and FAF were contributed by Hb & HCT, TG &HDL-C, ALT & GGT, and NAFLD & BMI in both males and females, standing for erythrocyte viscosity, lipid metabolism, hepatic enzyme metabolism, and fat accumulation respectively. LVF stood for lipid viscosity with inclusive (i.e. high loadings in factor analysis) of TC & LDL-C in both genders, and with further inclusive of HDL-C in females BPF reflected SBP & DBP in both genders, while BMI also had high loading on this factor in females. GMF, with FBG as the main manifestation, stood for glucose metabolism status, while SUA & CREA also had high loadings in males, and WBC count in females. This finding suggests that serum glucose concentration may be involved in renal function and inflammatory response. Similarly, IRF stood for inflammation response status with WBC count as the key element, clustered together with CREA in males while SUA & CREA in females, suggesting that WBC count was related to inflammation response status, which may link with renal function.

### Synthetic predictor and its application

In CVD prevention, a well-established primary healthcare strategy is to identify population at higher risk using prediction models in order to prevent CVD at earlier stages [48]. Several prediction models, including the Framingham risk score, have been applied in healthcare and management in different populations [49-54]. However, the predictive powers of these models are relatively low (AUC usually around 0.80), which may be partially due to the limited number of selected predictive factors in these models. To improve and optimize the predictive models, one of the methods is to identify and add new predictors in the predictive models. In China, as part of primary healthcare support, routine health check-up for urban residents has been developed rapidly in recent years. A number of convenient and inexpensive measurements for CVD and diabetes related biomarkers are included in the health check-up. It provides us with a unique opportunity to develop new predictive models using these biomarkers to extend the concept of MetS in a cost-effective manner. In our present study, a weighted *SP* was created by summarizing eight latent factors. The results show that *SP* was not only a good discriminative index (assessed by ROC curves, Figures 1_A1 and 1_B1), but also a significantly improved predictor for CHD (Figures 1_A2 and 1_B2). The AUC for the prediction of 5-year risk of CHD in the total study population was more than 0.85 in both genders (Figures 1_A2 and 1_B2) and demonstrated the *SP* could be used as a simple and effective health management tool in routine health check-up to predict subjects at high risk of CHD. Furthermore, *SP*-based 5-year CHD absolute risk matrix and relative absolute risk matrix can be easily applied in practice (Figure 2). For example, for a male at a given age who receives health check-up, the matrices show his absolute hazard (A1) and the relative hazard ratio (A2) as compared with the average hazard in the same age group in males.

### Advantages and limitations

The present study has several strengths. First, findings from the study were based on a large population-based sample from routine health check-up. People who receive the health check-up consist of the majority of urban Chinese employees, as they receive full coverage for healthcare supported by the Chinese government. Second, a weighted *SP* was developed using factor analysis from 8 latent factors of 16 measured variables, which represents eight metabolic-related factors (i.e., EVF, LVF, LMF, BPF, HEF, GMF, FAF, and IRF). This approach has the advantage of taking into consideration the associations between predictors and outcomes (i.e., the use of weighting method), rather than simply summing up the number of predictors [55]. Meanwhile, two main limitations should be kept in mind when interpreting the results. First, although the study included a large population-based sample size, the results cannot be generalized for the general population because the participants who received routine health check-ups are urban residents and are employed. Second, the time period of follow-up in this cohort analysis is relatively short. Therefore, further studies are needed to confirm the findings.

Despite the aforementioned limitations above, the present study, using data from routine health check-ups, extends the previous concept of MetS by including variables related to the MetS network that are ready to use from routine health check-up. The developed *SP* shows as a simple and validate predictor for identifying subjects at high risk of CHD.

## Supporting Information

Figure S1 **The combined proportion of the 4 basic components between male and female metabolic syndrome groups.** OB, obesity; HT, hypertension; HG, hyperglycemia; DY, dyslipidemia.
(TIF)

Table S1 **The incidence of coronary heart disease by follow-up year.**
(DOC)

Table S2 **Correlation matrix of sixteen biomarkers.**
(DOC)

Table S3 **The prevalence of the 4 basic components for both male and female metabolic syndrome groups.**
(DOC)

Table S4 **The standardized scoring coefficients of each factor for males and females.**
(DOC)
