# Supplementary figures and images for: Identification of Cardiovascular Risk Components in Urban Chinese with Metabolic Syndrome and Application to Coronary Heart Disease Prediction: A Longitudinal Study
Source: PLoS One. 2013 Dec 17;8(12):e84204. doi: 10.1371/journal.pone.0084204 (PMC3866125; doi:10.1371/journal.pone.0084204)

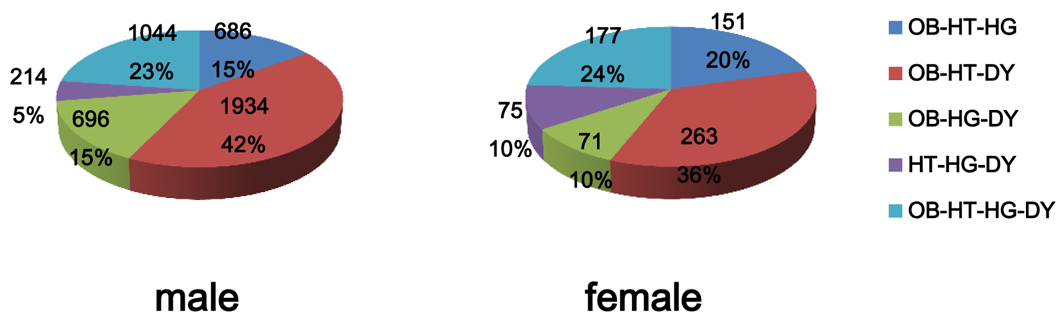

Supplement: Figure S1 — The combined proportion of the 4 basic components between male and female metabolic syndrome groups. OB, obesity; HT, hypertension; HG, hyperglycemia; DY, dyslipidemia. (TIF) [file pone.0084204.s001.tif]
