# Supplementary material for: Identification of Cardiovascular Risk Components in Urban Chinese with Metabolic Syndrome and Application to Coronary Heart Disease Prediction: A Longitudinal Study
Source: PLoS One. 2013 Dec 17;8(12):e84204. doi: 10.1371/journal.pone.0084204 (PMC3866125; doi:10.1371/journal.pone.0084204)
Supplement: Table S1 — The incidence of coronary heart disease by follow-up year. (DOC) [file pone.0084204.s002.doc]

**Table S1 The incidence of coronary heart disease by follow-up year.**

| Gender | 2005 | 2006 | 2007 | 2008 | 2009 | 2010 | cumulative incidence rate (%) |
| --- | --- | --- | --- | --- | --- | --- | --- |
| Male (n=1263) | 0 | 17 | 35 | 13 | 12 | 13 | 7.13 |
| Female (n=660) | 0 | 5 | 18 | 8 | 8 | 5 | 6.67 |
