# Supplementary material for: Identification of Cardiovascular Risk Components in Urban Chinese with Metabolic Syndrome and Application to Coronary Heart Disease Prediction: A Longitudinal Study
Source: PLoS One. 2013 Dec 17;8(12):e84204. doi: 10.1371/journal.pone.0084204 (PMC3866125; doi:10.1371/journal.pone.0084204)
Supplement: Table S2 — Correlation matrix of sixteen biomarkers. (DOC) [file pone.0084204.s003.doc]

**Table S2 Correlation matrix of sixteen biomarkers.**

|  | FLD | BMI | SBP | DBP | UA | FBG | CHOL | TG | HDL-C | LDL-C | WBC | ALT | GGT | CREA | Hb | HCT |
| --- | --- | --- | --- | --- | --- | --- | --- | --- | --- | --- | --- | --- | --- | --- | --- | --- |
| FLD | 1 | 0.135^*^ | -0.024 | 0.046^*^ | 0.094^*^ | 0.004 | 0.019 | 0.081^*^ | -0.036^*^ | 0.006 | 0.016 | 0.068^*^ | 0.044^*^ | -0.023^*^ | 0.077^*^ | 0.063^*^ |
| BMI |  | 1 | 0.072^*^ | 0.162^*^ | 0.097^*^ | -0.010 | -0.033^*^ | 0.014 | -0.074^*^ | -0.013 | 0.085^*^ | 0.151^*^ | 0.062^*^ | -0.013 | 0.045^*^ | 0.063^*^ |
| SBP |  |  | 1 | 0.445^*^ | -0.044^*^ | 0.031^*^ | 0.050^*^ | -0.082^*^ | 0.092^*^ | 0.028^*^ | 0.046^*^ | -0.068^*^ | -0.045^*^ | 0.019 | -0.068^*^ | -0.071^*^ |
| DBP |  |  |  | 1 | 0.119^*^ | -0.090^*^ | 0.035^*^ | 0.070^*^ | 0.071^*^ | -0.003 | 0.031^*^ | 0.122^*^ | 0.172^*^ | 0.043^*^ | 0.270^*^ | 0.234^*^ |
| UA |  |  |  |  | 1 | -0.222^*^ | -0.002 | 0.142^*^ | -0.040^*^ | -0.020 | 0.060^*^ | 0.114^*^ | 0.188^*^ | 0.294^*^ | 0.189^*^ | 0.157^*^ |
| FBG |  |  |  |  |  | 1 | 0.129^*^ | 0.119^*^ | 0.101^*^ | 0.061^*^ | 0.036^*^ | 0.035^*^ | 0.020 | -0.073^*^ | 0.014 | -0.012 |
| CHOL |  |  |  |  |  |  | 1 | 0.289^*^ | 0.506^*^ | 0.789^*^ | 0.030^*^ | 0.067^*^ | 0.170^*^ | -0.032^*^ | 0.051^*^ | 0.071^*^ |
| TG |  |  |  |  |  |  |  | 1 | 0.353^*^ | -0.104^*^ | 0.064^*^ | 0.110^*^ | 0.237^*^ | -0.010 | 0.105^*^ | 0.078^*^ |
| HDL-C |  |  |  |  |  |  |  |  | 1 | 0.166^*^ | -0.041^*^ | -0.023 | 0.190^*^ | -0.066^*^ | -0.037^*^ | -0.025 |
| LDL-C |  |  |  |  |  |  |  |  |  | 1 | 0.042^*^ | 0.061^*^ | 0.062^*^ | -0.004 | 0.061^*^ | 0.079^*^ |
| WBC |  |  |  |  |  |  |  |  |  |  | 1 | 0.042^*^ | 0.025 | 0.093^*^ | 0.125^*^ | 0.175^*^ |
| ALT |  |  |  |  |  |  |  |  |  |  |  | 1 | 0.415^*^ | -0.018 | 0.231^*^ | 0.198^*^ |
| GGT |  |  |  |  |  |  |  |  |  |  |  |  | 1 | -0.019 | 0.197^*^ | 0.187^*^ |
| CREA |  |  |  |  |  |  |  |  |  |  |  |  |  | 1 | 0.149^*^ | 0.144^*^ |
| Hb |  |  |  |  |  |  |  |  |  |  |  |  |  |  | 1 | 0.909^*^ |
| HCT |  |  |  |  |  |  |  |  |  |  |  |  |  |  |  | 1 |

* P≤0.05
