# Supplementary material for: Identification of Cardiovascular Risk Components in Urban Chinese with Metabolic Syndrome and Application to Coronary Heart Disease Prediction: A Longitudinal Study
Source: PLoS One. 2013 Dec 17;8(12):e84204. doi: 10.1371/journal.pone.0084204 (PMC3866125; doi:10.1371/journal.pone.0084204)
Supplement: Table S3 — The prevalence of the 4 basic components for both male and female metabolic syndrome groups. (DOC) [file pone.0084204.s004.doc]

**Table S3 The prevalence of the 4 basic components for both male and female metabolic syndrome groups.**

| Component | Male | | Female | | *χ^2^* | *P* |
| --- | --- | --- | --- | --- | --- | --- |
|  | n/N | % | n/N | % |  |  |
| Obesity | 4360/4574 | 95.32 | 662/737 | 89.82 | 37.29 | <0.001 |
| Hypertension | 3878/4574 | 84.78 | 666/737 | 90.37 | 16.01 | <0.001 |
| Hyperglycemia | 2640/4574 | 57.72 | 474/737 | 64.31 | 11.39 | <0.001 |
| Dyslipidemia | 3888/4574 | 85.00 | 586/737 | 79.51 | 14.41 | <0.001 |
