# Supplementary material for: Identification of Cardiovascular Risk Components in Urban Chinese with Metabolic Syndrome and Application to Coronary Heart Disease Prediction: A Longitudinal Study
Source: PLoS One. 2013 Dec 17;8(12):e84204. doi: 10.1371/journal.pone.0084204 (PMC3866125; doi:10.1371/journal.pone.0084204)
Supplement: Table S4 — The standardized scoring coefficients of each factor for males and females. (DOC) [file pone.0084204.s005.doc]

**Table S4** **The standardized scoring coefficients of each factor for males and females.**

|  | **Male** (n=4574) | | | | | | | | **Female** (n=737) | | | | | | | |
| --- | --- | --- | --- | --- | --- | --- | --- | --- | --- | --- | --- | --- | --- | --- | --- | --- |
|  | Factor1 | Factor2 | Factor3 | Factor4 | Factor5 | Factor6 | Factor7 | Factor8 | Factor1 | Factor2 | Factor3 | Factor4 | Factor5 | Factor6 | Factor7 | Factor8 |
|  | EVF | LVF | LMF | BPF | HEF | GMF | FAF | IRF | LVF | EVF | HEF | BPF | IRF | LMF | GMF | FAF |
| NAFLD | 0.029 | 0.031 | 0.090 | -0.069 | -0.210 | 0.049 | 0.757 | -0.144 | 0.033 | 0.036 | -0.094 | -0.097 | -0.014 | 0.050 | -0.030 | 0.788 |
| BMI | -0.110 | 0.019 | -0.149 | 0.105 | 0.182 | -0.064 | 0.483 | 0.171 | -0.006 | -0.106 | 0.077 | 0.276 | -0.008 | -0.044 | 0.114 | 0.416 |
| SBP | -0.085 | 0.005 | -0.024 | 0.596 | -0.077 | -0.079 | -0.033 | 0.059 | 0.087 | -0.049 | -0.038 | 0.508 | 0.042 | -0.097 | 0.117 | -0.041 |
| DBP | 0.063 | -0.039 | 0.024 | 0.527 | 0.036 | 0.053 | 0.026 | -0.101 | -0.070 | 0.035 | 0.028 | 0.546 | -0.070 | 0.111 | -0.209 | -0.027 |
| SUA | -0.038 | -0.012 | 0.110 | -0.045 | 0.064 | 0.558 | 0.087 | 0.117 | 0.008 | 0.063 | 0.039 | -0.061 | 0.556 | -0.027 | -0.158 | 0.123 |
| FBG | -0.061 | -0.009 | 0.109 | -0.001 | 0.034 | -0.576 | 0.049 | 0.236 | 0.031 | -0.051 | -0.006 | -0.078 | -0.133 | -0.061 | 0.762 | 0.056 |
| Total cholesterol | -0.025 | 0.461 | 0.122 | -0.012 | -0.025 | -0.002 | 0.023 | 0.000 | 0.451 | -0.037 | -0.024 | 0.010 | 0.007 | 0.079 | -0.020 | 0.029 |
| Triglycerides | 0.009 | -0.173 | 0.564 | -0.073 | -0.012 | -0.005 | 0.084 | 0.104 | -0.137 | -0.023 | -0.022 | -0.053 | 0.011 | 0.756 | -0.021 | 0.097 |
| HDL-C | -0.020 | 0.068 | 0.450 | 0.085 | -0.086 | -0.005 | -0.104 | -0.093 | 0.152 | -0.032 | 0.012 | 0.098 | -0.036 | 0.417 | -0.027 | -0.140 |
| LDL-C | -0.025 | 0.584 | -0.218 | -0.025 | 0.006 | 0.004 | 0.034 | -0.010 | 0.512 | -0.041 | -0.001 | -0.021 | 0.035 | -0.237 | -0.026 | 0.050 |
| WBC count | 0.031 | -0.043 | 0.020 | -0.033 | 0.025 | -0.145 | 0.041 | 0.716 | -0.145 | 0.069 | 0.023 | 0.102 | 0.284 | 0.072 | 0.448 | -0.059 |
| ALT | -0.034 | -0.006 | -0.149 | -0.044 | 0.611 | -0.036 | -0.036 | 0.036 | -0.003 | -0.008 | 0.539 | -0.006 | -0.027 | -0.035 | -0.040 | 0.054 |
| GGT | -0.049 | -0.017 | 0.099 | 0.005 | 0.506 | 0.062 | -0.118 | -0.078 | -0.022 | -0.100 | 0.604 | 0.030 | 0.012 | 0.006 | 0.054 | -0.139 |
| Serum creatinine | -0.066 | 0.058 | 0.006 | 0.024 | -0.117 | 0.288 | -0.167 | 0.491 | 0.048 | -0.067 | -0.052 | 0.015 | 0.529 | -0.005 | 0.054 | -0.115 |
| Hemoglobin | 0.508 | -0.032 | 0.001 | -0.012 | -0.039 | 0.006 | -0.029 | -0.034 | -0.047 | 0.515 | -0.067 | -0.027 | 0.007 | -0.016 | -0.022 | 0.006 |
| Hematokrit | 0.508 | -0.020 | -0.008 | -0.030 | -0.058 | 0.012 | -0.035 | 0.017 | -0.039 | 0.503 | -0.031 | -0.023 | -0.005 | -0.039 | -0.014 | -0.015 |

Factors were named as erythrocyte viscosity factor (EVF), lipid viscosity factor (LVF), lipid metabolism factor (LMF), blood pressure factor (BPF), hepatic enzyme factor (HEF), glucose metabolism factor (GMF), fat accumulation factor (FAF), and inflammation response factor (IRF). NAFLD indicates non-alcoholic fatty liver; BMI, body mass index; SBP, systolic blood pressure; DBP, diastolic blood pressure; SUA, serum uric acid; FBG, fasting blood-glucose; HDL-C, high-density lipoprotein cholesterol; LDL-C, low-density lipoprotein cholesterol; WBC, white blood cell; ALT, alanine aminotransferase; and GGT, gamma-glutamyl transpeptidase.
